# Performance of a Vendor System for Systematic Offline Adaptive Breast Helical Radiotherapy

**DOI:** 10.3390/cancers18091386

**Published:** 2026-04-27

**Authors:** Philippe Meyer, Claire Dossun, Georges Noel, Loris Barrier, Anthony Richert, Florence Arbor, Claudine Niederst

**Affiliations:** 1 Department of Radiation Therapy, Centre Paul Strauss, 3 Rue de la Porte de l’Hôpital, 67000 Strasbourg, France; c.dossun@institut-strauss.fr (C.D.); a.richert@institut-strauss.fr (A.R.); f.arbor@institut-strauss.fr (F.A.); c.niederst@institut-strauss.fr (C.N.); 2 Team IMAGeS, ICube Laboratory, University of Strasbourg, 67412 Illkirch, France

**Keywords:** breast cancer, adaptive radiotherapy (ART), deformable registration (DIR), image-guided radiotherapy (IGRT)

## Abstract

**Background:** This study evaluated the performance of a commercial offline adaptive radiotherapy system for systematic monitoring of breast cancer treatment with nodal irradiation using helical tomotherapy. **Methods:** Thirty patients treated for invasive unilateral breast carcinoma were analysed. For each patient, three megavoltage CT scans acquired at the first, middle, and last treatment sessions were processed through the PreciseART (Accuray, US) offline ART workflow. Automatically deformed structures were compared with manually delineated reference structures. Geometric accuracy was assessed using the Dice similarity coefficient (DSC), Hausdorff distance (HD95), mean distance to agreement (MDA), and barycentre distance (BD). The dosimetric parameters included D2% and V95% for targets and Dmean/Dmax/V20Gy for organs at risk. **Results:** Median DSCs exceeded 0.9 for the CTVbreast, PTVbreast, heart, and ipsilateral lung and were above 0.8 for the remaining structures, except the CTVn and oesophagus. Dosimetric differences between deformed and reference structures were within 5% for D2% across all targets and for V95% of the CTVbreast and PTVbreast in 90% of the sessions. The ipsilateral lung V20Gy differed by less than 5% in more than 90% of the sessions. Larger deviations (up to 10%) were observed for the nodal PTVs and mean heart dose, while the greatest inconsistencies were found for the oesophagus and spinal canal. **Conclusions:** The evaluated offline ART system demonstrates sufficient accuracy for automated monitoring of breast and lung structures. However, cautious interpretation remains necessary for nodal targets, heart, and oesophagus dosimetry prior to clinical implementation.

## Simple Summary

This study addresses the growing need for reliable adaptive radiotherapy (ART) tools to monitor anatomical and dosimetric changes during complex breast cancer treatments that include nodal irradiation. The objective was to evaluate whether a commercial offline ART system can accurately reproduce target volumes and organs at risk throughout the treatment course, thereby supporting safe and efficient automated monitoring. By comparing automatically propagated contours with expert manual delineations, geometric accuracy and dosimetric consistency were assessed. The results show that the system performs satisfactorily for the primary breast target and lung structures while demonstrating notable limitations in the delineation of nodal targets and certain critical organs, particularly the heart and oesophagus. These findings provide evidence-based insights into the current strengths and limitations of offline ART systems, highlighting areas of robustness as well as aspects that require further methodological refinement. Ultimately, this work may help inform future technological improvements and guide the cautious clinical adoption of offline ART in breast cancer radiotherapy.

## 1. Introduction

Daily imaging in adaptive radiotherapy (ART) allows treatment plans to be adjusted according to patients’ anatomical changes. Although the concept of ART was first introduced in 1997 [1], its clinical use remained limited for many years due to technological limitations [2]. Over the past decade, advances in imaging, computing, and AI have enabled routine ART implementation using MRI- or CBCT-guided systems [3].

ART techniques can be classified into offline and online approaches. Offline ART involves replanning between treatment sessions, whereas online ART performs replanning during the session with the patient on the table. Historically, offline ART relied on simple, periodic triggers (every X sessions, weekly, halfway through treatment) [4]. These methods lack individualization: rescheduling is applied to all patients indiscriminately and periodically, even though not all patients need ART, and some patients may require ART more frequently [5]. To be fully beneficial, the offline ART process must be able to calculate dosimetric indicators specific to each location and for each session so that rescheduling is triggered in a relevant manner [6].

ART is applicable to many anatomical locations [7]. In 2022, breast cancer was the most common cancer in women in terms of incidence and the second in terms of mortality in developed countries [8]. ART for breast cancer is a fully justified technique because of the anatomical changes or positioning difficulties that can occur during treatment. There are many causes for these changes, such as a decrease or increase in the tumour/oedema/lymphocele, changes in arm mobility or heart position, and weight loss/gain [9,10]. Few publications have reported offline ART trials for breast cancer treatment. One IMRT-SIB-based trial showed that rescanning patients with an initial seroma volume >30 cm^3^ shortly after lumpectomy significantly reduced high-dose volumes [11]. This strategy has been proven to lead to a significant reduction in high-dose volumes, but the dose recalculation process is entirely manual and does not include positioning imaging, and the criteria for selecting patients for rescanning remain limited.

To integrate offline ART into routine practice, automation of all key steps (image exports, segmentation, planning, data analysis) is essential. The first integrated offline ART system, PreciseART (Accuray, Madison, USA), was commercialized in 2018 for helical radiotherapy machines [12,13]. Once the session is finished, the system automatically recalculates the dose delivered on the daily positioning image, considering the patient’s anatomic variations. The planning CT contours are deformed and propagated to the daily positioning image, allowing automatic recalculation of the dose-volume histogram (DVH) so that the user can assess whether the delivered treatment is still in accordance with the planned treatment. A key limitation of this workflow is the reliability of the deformable image registration (DIR) used to propagate contours. For the DVHs recalculated on this imaging to be usable, the contours must be accurate. It has been shown qualitatively that, under certain conditions, the PreciseART DIR algorithm can produce aberrant results and therefore poor-quality contours [9]. However, if a systematic offline ART process is to be applied in routine clinical practice, it is difficult to imagine having to check and correct all contours for all patients after each treatment session.

The objective of our study was to evaluate a commercial offline ART system used for systematic monitoring of all treatment sessions in patients undergoing whole-breast irradiation with nodal involvement using helical tomotherapy. The performance of the DIR algorithm used to propagate contours onto the daily positioning image was quantified. To do this, the contours of 30 tomotherapy patients were manually delineated by a radiation therapist on positioning megavoltage computed tomography (MVCT) images at the first, mid-treatment, and last sessions. These contours were compared with those automatically generated by a commercial offline ART system, and their dosimetric impact was quantified. The aim was to determine whether it is possible to rely on the contours generated by an offline ART system without needing to check them individually. The objective was to use an automatic replanning trigger directly on the dosimetric metrics calculated on the daily positioning image, which would allow many patients to be monitored in routine clinical practice.

## 2. Materials and Methods

### 2.1. Patients

Thirty female patients were enrolled between September 2022 and April 2023 as part of the DIRB-ATOM monocentric observational study (clinicaltrials.gov, NCT05383144, date of registration 19 May 2022). Patients were treated with helical tomotherapy for invasive unilateral breast carcinoma requiring regional lymph node irradiation, including level I (lower axilla) and, when indicated, level II (upper axilla), III (infraclavicular), IV (supraclavicular), interpectoral (Rotter), and/or internal mammary chain (IMC) after mastectomy or breast-conserving surgery. Treatments consisted of either 25 fractions of 2 Gy or 15 fractions of 2.67 Gy, five days per week.

The patient’s kilovoltage computed tomography (kVCT) used to plan the treatment was acquired on a 120 kV GE Optima CT580 RT (General Electric, Chicago, IL, USA) with a 2.5 mm slice thickness. Patients were positioned supine with arms elevated, using a Monarch immobilization device (CQ Medical, Avondale, AZ, USA). The organs at risk (OAR), clinical target volumes (CTVs), and planned target volumes (PTVs) were manually segmented by the same radiation oncologist and included the CTVbreast, CTVn, PTVbreast, PTVn, heart, oesophagus, spinal canal, and ipsilateral lung. The delineation of the CTVbreast and CTVn followed the ESTRO recommendations [14], and those for organs at risk followed the practice of external radiotherapy and brachytherapy [15]. A 5 mm CTV-to-PTV margin was applied, and skin retraction was 0.3 cm in the absence of skin invasion. Treatments were planned with Precision 3.0.2 (Accuray, Madison, WI, USA) and Volo Classic devices, with a 5 cm field width, a pitch of 0.287, and a modulation factor between 2.5 and 3.0. Before each session, patient positioning was checked using MVCT imaging.

### 2.2. Study Design

**Figure 1:**
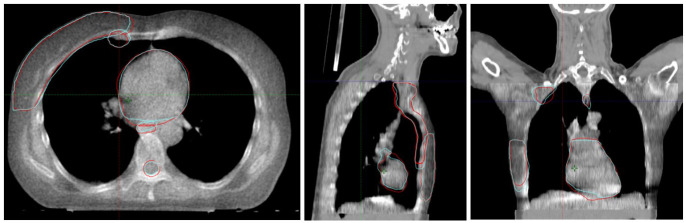
Transverse, sagittal, and coronal views of the merged image of a patient included in this study. Red: propagated structure from PreciseART (Struct_DIR_). Blue: manually delineated structure (Struct_REF_).

Patients were enrolled using the ART offline software PreciseART 7.1.101. The PreciseART workflow is triggered automatically after each treatment session [12]. A “merged” image is first created by rigidly registering the planning kVCT to extend the daily MVCT head-to-foot and beyond the MVCT field of view (FOV). The original treatment plan is then applied to this merged image using MVCT and kVCT Hounsfield unit-to-density curves inside and outside the MVCT FOV, respectively, to calculate the delivered dose for that fraction. A DIR is performed between the planning kVCT and the merged MVCT to deform the original structures onto the daily image. These structures are referred to as Struct_DIR_ (see Figure 1). The DIR algorithm uses a non-parametric non-rigid transformation [16].

**Figure 2:**
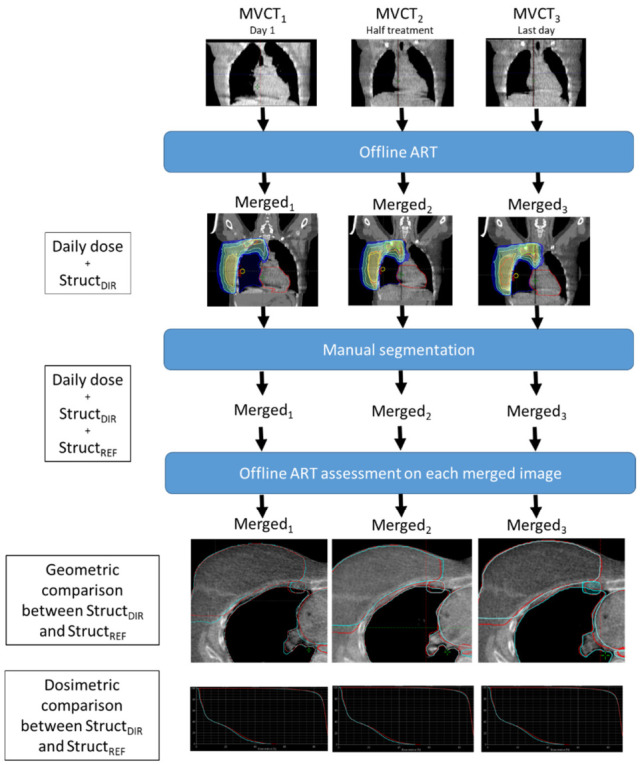
Study design. For each patient included, three MVCT positioning images were used: at the first session, in the middle of treatment, and at the end of treatment. These images were processed by a commercial offline ART system and segmented manually. A geometric and dosimetric evaluation of the offline ART system was performed.

The study design is summarized in Figure 2. For the purposes of the study, three MVCTs were analysed per patient: MVCT_1_ at the first session, MVCT_2_ at mid-treatment, and MVCT_3_ at the last session. The MVCT covered the entire target volume region, extending from the supraclavicular area to the diaphragm, and was acquired using the routine clinical parameters (coarse mode, 3 mm slice thickness). Each merged examination included a structure file (Struct_DIR_) derived from the deformable registration between the kVCT and MVCT examinations and a dose file derived from the recalculation of the dose distribution. The same clinician who contoured the planning CT also manually segmented all structures on each merged image using Eclipse v17 (Siemens Healthineers, Forchheim, Germany). These manual contours served as reference structures (Struct_REF_) and were cropped to the MVCT volume. Geometric and dosimetric metrics derived from Struct_DIR_ were compared with those from Struct_REF_ for each patient and each of the three sessions.

### 2.3. Geometric and Dosimetric Metrics

Each geometric and dosimetric metric was calculated 90 times (three sessions for each of the 30 patients). For geometric assessment, the Dice similarity coefficient (DSC), 95% Hausdorff distance (HD95) (rather than the Hausdorff distance to limit the influence of outliers), mean distance to agreement (MDA), and barycentre distance (BD) were calculated to assess matching between the Struct_DIR_ and Struct_REF_. The definitions of the metrics are given in Supplementary File S1. Each geometric metric was averaged for all patients by treatment stage (day 1, half-treatment, and last day). To assess the ART DIR performance, we compared it to published tolerance thresholds. AAPM TG-132 recommends DSC > 0.8 and MDA < 3 mm as acceptable limits [17]. We pragmatically set the threshold at ≥90% of sessions meeting the criteria for clinical robustness. To date, no published consensus has established tolerance thresholds for HD95 and BD; therefore, these metrics were interpreted by comparison with values reported in other studies.

With respect to the dosimetric metrics, the objective was to determine whether the dose delivered to the Struct_DIR_ and Struct_REF_ differed significantly. CTV and PTV coverage were defined as the percent target volume receiving 95% of the prescribed dose (V95%) and the dose received by 2% of the target volume (D2%), respectively. For OARs, the mean dose (Dmean) was calculated for the heart and oesophagus, the maximal dose (Dmax) was calculated for the spinal canal, and the percent volume receiving 17 or 20 Gy (V17Gy or V20Gy, depending on the prescription) was calculated for the ipsilateral lung. Given that the prescription may differ between two patients, the relative difference between Struct_DIR_ and Struct_REF_ was calculated. Relative differences were averaged across patients for each treatment stage (day 1, half-treatment, and last day). It is difficult to define a single meaningful dosimetric threshold to determine whether the value of this relative difference is clinically acceptable. Indeed, this depends on the organ, the dosimetric criterion considered, and the clinical context. Glide et al. proposed a dosimetric tolerance of ±5% during end-to-end testing of ART workflows [13]. We used this ±5% criterion, along with an additional threshold arbitrarily set at ±10%, and arbitrarily set the threshold for clinical acceptability at 90% of sessions meeting these criteria.

### 2.4. Statistical Analysis

For each structure and dosimetric metric, the values derived from Struct_DIR_ were compared with the reference values from Struct_REF_ using paired statistical tests. The normality of intra-patient difference distributions was assessed using the Shapiro–Wilk test. As most distributions were found to be non-normal (*p* < 0.05), the Wilcoxon signed-rank test was systematically applied to compare paired values. Clinical reliability was evaluated based on the percentage of patients showing a relative difference below tolerance thresholds of 5% and 10% (see Section 2.3). The statistical significance threshold was set at *p* < 0.05.

## 3. Results

All the numerical values are given in Supplementary File S2.

### 3.1. Geometric Metrics

**Figure 3:**
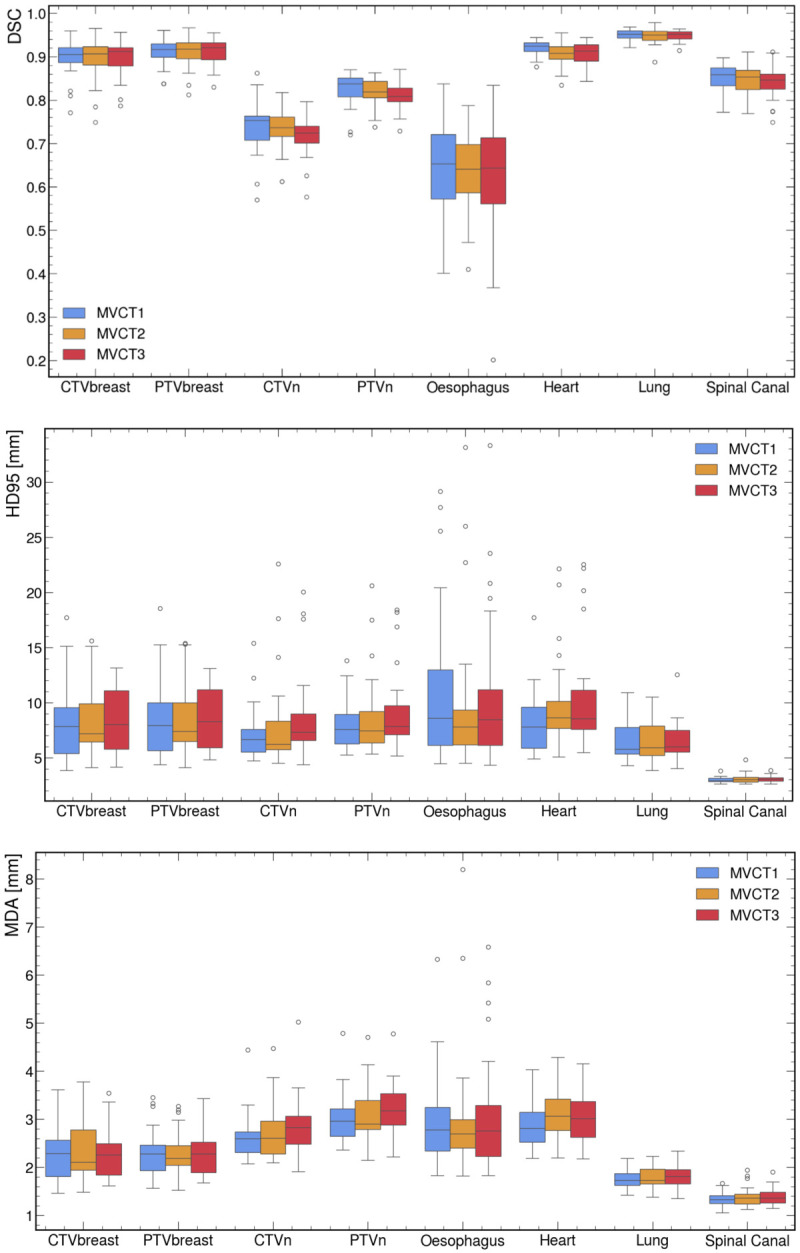

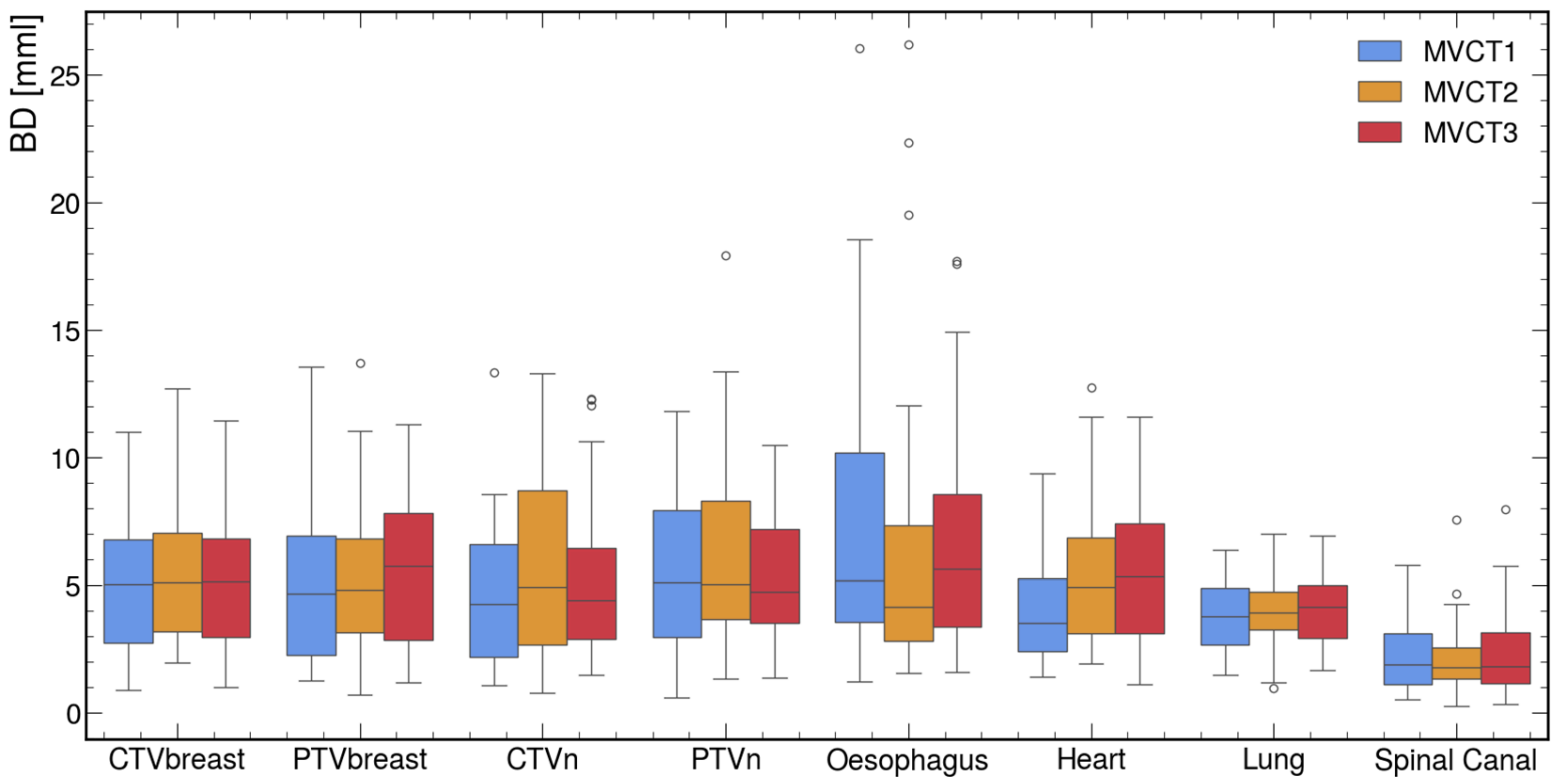
Box plots of geometric metrics comparing propagated structures (Struct_DIR_) and reference manual delineations (Struct_REF_) for all patients and treatment stages (first, mid-treatment, and last session). The evaluated metrics include the Dice similarity coefficient (DSC), 95% Hausdorff distance (HD95), mean distance to agreement (MDA), and barycentre distance (BD).

The results of the geometric metrics are shown for all the structures in a box plot in Figure 3. Overall, there was no significant difference between the values of the metrics calculated at the beginning (MVCT_1_), middle (MVCT_2_), and end (MVCT_3_) of treatment, regardless of the structure and geometric metric considered.

More specifically, for the OARs, the median DSCs were relatively high for all structures, greater than 0.9 for the heart and ipsilateral lung, and greater than 0.8 for the spinal canal. Only the oesophagus had a DSC less than 0.8. For the target volumes, the median DSC values were greater than 0.9 for the CTVbreast and PTVbreast and greater than 0.8 for the PTVn, with only the CTVn having a DSC less than 0.8. However, it should be noted that the DSC for the PTVn was less than 0.8 for approximately one-quarter of the sessions. The median MDAs ranged from 1.5 to 3 mm, depending on the structure considered, with values greater than 3 mm for approximately 50% of the sessions for the PTVn, oesophagus, and heart. The median HD95 was approximately 7 to 8 mm for all structures, except the lung and spinal canal, for which it was 5.9 and 3.0 mm, respectively. The median BD was approximately 5 mm for all structures, except the lung and spinal canal, for which it was 3.9 and 1.8 mm, respectively.

Table 1 shows the percentage of sessions for which AAPM TG-132 tolerances are met for DSC and MDA, per structure. We found that the DSC performance of the DIR algorithm used in PreciseART met AAPM tolerances for the structures included in this study in more than 90% of the sessions, except for the CTVn, PTVn, spinal canal, and oesophagus. The MDA values we obtained met AAPM tolerances for all structures in more than 90% of the sessions, except for the CTVn, PTVn, oesophagus, and heart.

### 3.2. Dosimetric Metrics

**Figure 4:**
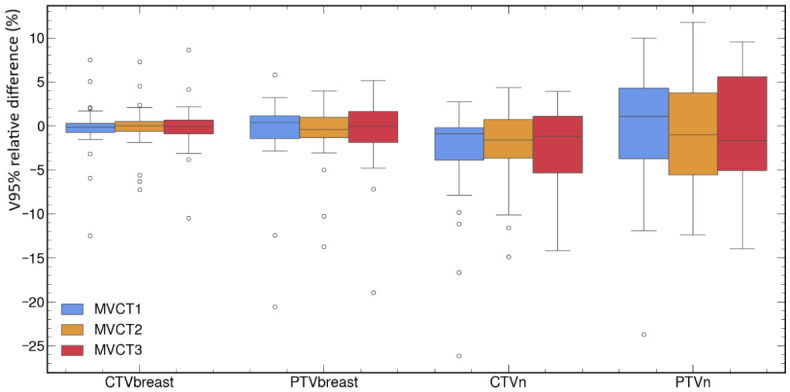

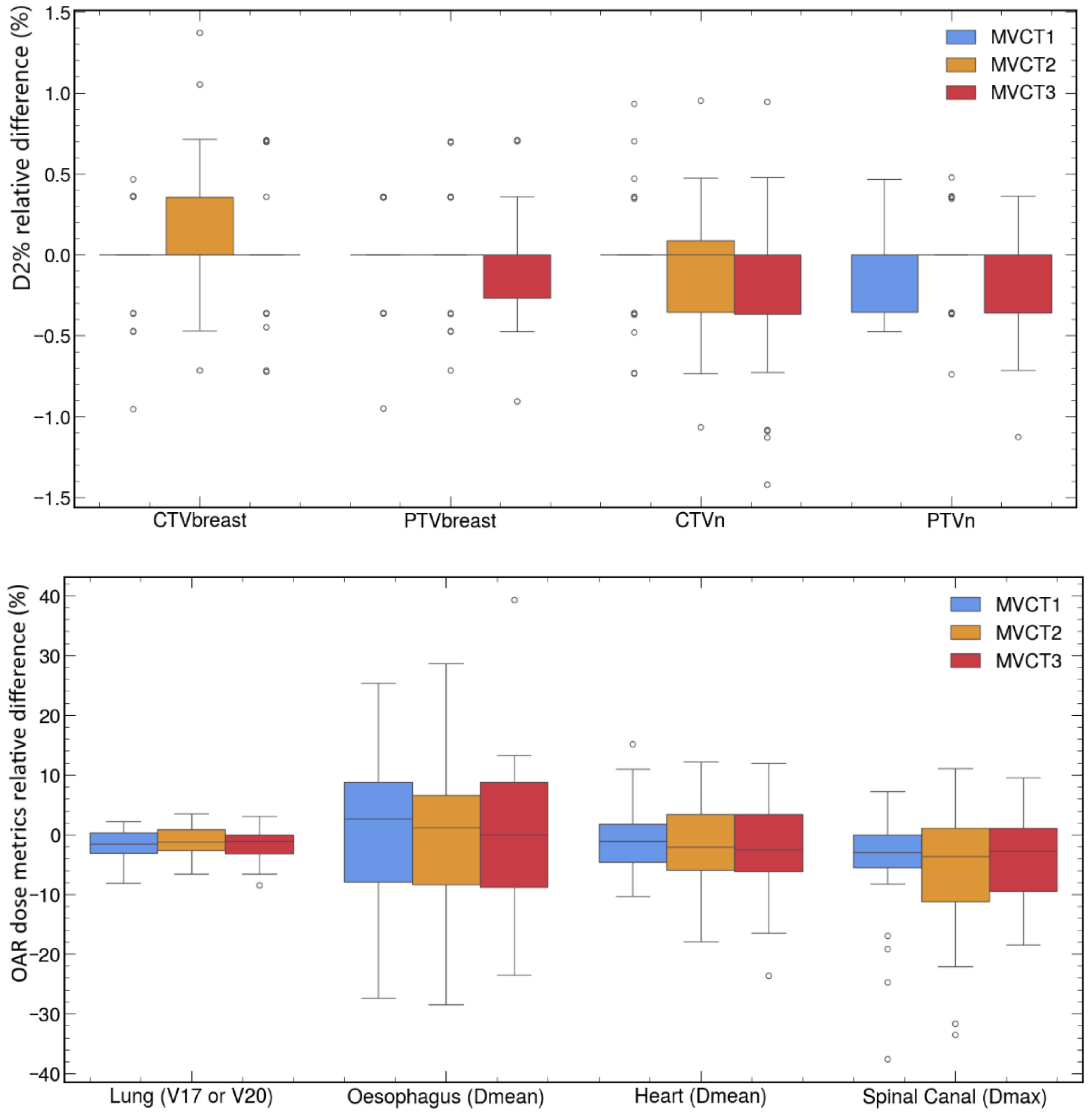
Box plots of relative dosimetric differences (%) between propagated structures (Struct_DIR_) and reference manual delineations (Struct_REF_) for all patients and treatment stages (first, mid-treatment, and last session). Target volume metrics include V95% (volume receiving ≥95% of the prescribed dose) and D2% (dose received by 2% of the volume). Organ-at-risk (OAR) metrics include Dmean for the heart and oesophagus, Dmax for the spinal canal, and V17Gy/V20Gy for the ipsilateral lung, depending on the prescription.

Relative differences between Struct_DIR_ and Struct_REF_ dosimetric metrics are shown for all the structures in a box plot in Figure 4. As with the geometric metrics, there was no significant difference between the values of the metrics calculated at the beginning (MVCT_1_), middle (MVCT_2_), and end (MVCT_3_) of treatment, regardless of the structure and dosimetric metric considered. The *p*-value calculated to compare Struct_DIR_ and Struct_REF_ dosimetric metrics, as well as the percentage of sessions for which the relative dosimetric differences we observed met a tolerance of ±5% or ±10%, are given in Table 2.

Our results show that the median deviation between the dosimetric metrics calculated based on Struct_DIR_ and Struct_REF_ varied between 0% and 3%, depending on the structure, but with high standard deviations, indicating significant disparities between patients and sessions. Concerning target volumes, we observed that the median difference for the D2% was close to 0%, with all differences within ±5% for all the sessions. Therefore, the D2% values for the target volumes were very similar, whether they were calculated on the structures delineated by the physician or on those deformed by the PreciseART algorithm. The same observation can be made for the V95% of the CTVbreast and PTVbreast: the median deviation between the V95% calculated for the Struct_DIR_ and Struct_REF_ was less than 1%, with approximately 90% of deviations within ±5%. For lymph node target volumes, the results are more varied: although the median difference was less than 2%, the dosimetric deviation between the V95% of the AI-deformed PTVn or CTVn and that achieved by the physician exceeded ±5% for 49% and 23% of the sessions, respectively. Notably, this difference remained within ±10% for nearly 90% of the sessions.

With respect to OARs, the median maximum dose to the spinal canal was approximately 3% greater for the contour drawn manually by the physician than for the deformed contour, with 51% and 26% of sessions exceeding deviations of ±5% and ±10%, respectively. For the ipsilateral lung, the median V17 or V20Gy differed by 1% between the contour manually drawn by the physician and the deformed contour, with more than 90% of sessions having deviations within ±5%. The median average dose to the heart was approximately 2% greater for the physician’s contour than for the deformed contour, with 42% and 16% of sessions having deviations exceeding ±5% and ±10%, respectively. The deviations between the average dose to the oesophagus calculated on the Struct_DIR_ and Struct_REF_ exceeded ±5% and ±10% for 68% and 43% of the sessions, respectively.

Finally, it should be noted that the relevance of the Wilcoxon test remains clinically limited. For example, there is no significant directional bias between the oesophagus mean dose calculated on Struct_DIR_ and Struct_REF_; however, it is the least reliable structure in the study, with high variability reflected by only 32% of sessions showing deviations within ±5% and 57% within ±10%. Conversely, the V17/V20 lung metric showed a statistically significant difference (*p* = 0.001), yet remained clinically negligible (within ±5% deviation in 89% of sessions).

## 4. Discussion

In this study, we evaluated commercial offline ART software with the aim of determining whether it can be used routinely in clinical practice for breast cancer with lymph node involvement, without requiring systematic verification of the automatically generated contours. The performance of the DIR algorithm used to generate target volumes and OARs on MVCT positioning images was quantified geometrically and dosimetrically in 30 patients, with three sessions each.

Other teams have evaluated PreciseART on head-and-neck and cervical cancer patients [18,19,20,21], but this study is the first to quantify the performance of PreciseART or any other DIR propagation algorithms on MVCT for breast cancer treatment. Comparable data from CBCT-based ART studies provide useful context. Using geometric metrics, Liang et al. reported average DSCs of 0.89, 0.90, and 0.76 for the breast CTV, heart, and spinal canal, respectively [22]. A vendor online ART system combining deformation algorithms and deep learning achieved DSCs of 0.92, 0.93, and 0.96 and HD95 of approximately 5 mm, 8.8 mm, and 5.5 mm for the breast CTV, heart, and lung, respectively [23]. Still using CBCT but with deep learning-based structure segmentation, Wang et al. compared different training strategies, achieving DSCs of 0.84, 0.86, and 0.80 and HD95 of 8.8 mm, 3.74 mm, and 4.29 mm for the breast CTV, heart, and spinal canal, respectively [24]. Another 3D U-Net-based algorithm evaluated on synthetic pseudo-CT images derived from CBCT images achieved DSCs of 0.83, 0.90, and 0.81 and HD95 of 10.8 mm, 9.3 mm, and 2.3 mm for the breast CTV, heart, and spinal canal, respectively [25]. Our study revealed that PreciseART achieves average DSCs of 0.90, 0.91, 0.95, and 0.85 and HD95 of 8.25, 9.34, 6.52, and 3.06 mm for the breast CTV, heart, lung, and spinal canal, respectively. For these structures, its performance appears at least comparable to published CBCT-based algorithms.

However, geometric metrics alone may not be sufficient to predict the clinical relevance of automatic segmentation algorithms, and dosimetric metrics are preferable [26,27]. No prior studies have quantified the dosimetric performance of PreciseART or other DIR propagation algorithms on MVCT for breast cancer. Almberg et al. evaluated the dosimetric impact of an automatic segmentation model on kVCT on different structures for breast cancer treatment, reporting significant differences for some metrics but deeming them clinically irrelevant [28]. However, these results should be interpreted cautiously, as they are based on small average deviations and do not report individual variability, which may be clinically significant. A similar study comparing an AI-based segmentation algorithm with manual contours also reported small average differences across OAR dosimetric metrics, but again without detailing patient-specific outliers [29]. In both studies, the standard deviations exceeded the mean deviations, suggesting substantial variability for certain patients. Our results are consistent with these observations: the median deviation between the dosimetric metrics calculated based on the Struct_DIR_ and Struct_REF_ was less than 5% in all cases, but the deviation could be much greater for individual patients.

Overall, the D2% of the target volumes propagated by PreciseART was reliable across sessions. The confidence level of the V95% of the CTVbreast and PTVbreast and the V17/V20 Gy of the ipsilateral lung was also high, with a maximum dosimetric deviation of 5% for at least 90% of the sessions. With a lower confidence level (maximum dosimetric deviation of 10%), the V95% of the PTVn and CTVn and the average dose to the heart could only be trusted in 85% of the sessions, highlighting the need for manual review. The maximum spinal canal dose and mean oesophageal dose showed limited reliability, although oesophageal uncertainty likely reflects Struct_REF_ variability rather than DIR performance. It should be noted that these results are specific to our clinical practice and that such an evaluation, including comprehensive end-to-end testing, should be conducted by each user [30].

A limitation of the study is the use of a single manual contour as the ground truth, despite the well-known inter- and intraobserver variability in radiotherapy segmentation [31]. For breast treatment, kVCT assessments have shown up to 22% and 35% variation in cardiac and breast volume, respectively, among nine observers [32]; a DSC of 0.94 for the heart among six observers [33]; or DSCs of 0.91, 0.98, 0.78, 0.69, and 0.85 for the heart, lungs, oesophagus, spinal canal, and breast CTV, respectively, among three observers [29]. Since, to our knowledge, there is no assessment of inter- and intraobserver contouring variability on MVCT for breast cancer treatment, we conducted an exploratory analysis as part of this study. By comparing the Struct_REF_ used in this study for two patients with those performed by another physician (interobserver variability), we found mean DSCs of 0.85, 0.99, 0.64, 0.83, 0.92, and 0.76 for the heart, lungs, oesophagus, spinal canal, PTV, and breast, respectively. For intraobserver variability, one physician recontoured MVCTs from two patients three times at one-week intervals; mean DSCs were 0.90, 0.99, 0.65, 0.84, 0.92, and 0.81 for the same structures. These values align with those obtained in the literature for kVCT imaging. When compared with the performance metrics obtained for PreciseART (see Table 3), the results appear broadly similar; however, given that the analysis is limited to two patients, no robust conclusions can be drawn, though the commercial DIR algorithm results remain within a plausible range.

Another limitation is the use of MVCT imaging for both the DIR algorithm and manual contouring, even though tomotherapy systems now offer kVCT imaging [34]. Maneepan et al. reported that PreciseART can be applied with kVCT in clinical head-and-neck cancer treatment to trigger adaptive replanning, but they did not assess deformation accuracy [35]. A phantom study comparing the two modalities reported that kVCT did not significantly improve DSC or MDA values over MVCT once measurement uncertainties were considered [36]. It also showed that kVCT images were less uniform, suggesting that MVCT remains a valuable option for ART. However, MVCT has two practical drawbacks that affect systematic offline ART. First, acquisition time is long, approximately 200 s for a breast with lymph nodes in coarse mode. In routine practice at our centre, therapists perform a complete head-to-feet acquisition of the volume to be treated during the first session, followed by a partial acquisition of this volume on a weekly basis during subsequent sessions. Implementing systematic offline ART would require full scans at every session, increasing treatment time. Second, MVCT has a limited 39 cm FOV, which can lead to faulty image reconstruction and inaccurate dose calculation, potentially causing unjustified alerts in an offline ART process [9].

## 5. Conclusions

The performance of the evaluated commercial offline ART system for breast cancer treatment with nodal irradiation is structure-dependent. It demonstrates sufficient accuracy for automated monitoring of breast target volumes (CTVbreast, PTVbreast) and the ipsilateral lung, with dosimetric deviations generally within ±5%. For nodal target volumes (CTVn, PTVn) and the heart, variability is too high for reliable unsupervised use, and systematic manual review is required. For the oesophagus and spinal canal, discrepancies are substantial and frequent, rendering the system unsuitable for clinical decision-making on these structures. Overall, the system is suitable for selective dose monitoring but not for guiding clinical decisions without structure-specific validation and manual verification where uncertainties are significant.

## Figures and Tables

**Table 1:** Percentage of sessions for which geometric criteria were met, by structure.

Structure	DSC > 0.8 (%)	MDA < 3 mm (%)
CTVbreast	96	88
PTVbreast	100	91
CTVn	7	77
PTVn	73	48
Oesophagus	3	68
Heart	100	53
Lung	100	100
Spinal Canal	89	100

**Table 2:** *p*-value calculated to compare Struct_DIR_ and Struct_REF_ dosimetric metrics and percentage of sessions for which dosimetric criteria were met, depending on the structure.

Structure	Metric	*p*-Value	% within ±5%	% within ±10%
CTVbreast	D2%	0.37	100	100
CTVbreast	V95%	0.28	90	98
PTVbreast	D2%	0.67	100	100
PTVbreast	V95%	0.57	90	94
CTVn	D2%	0.14	100	100
CTVn	V95%	<0.001	77	91
PTVn	D2%	0.03	100	100
PTVn	V95%	0.87	51	89
Heart	Dmean	0.05	58	84
Lung	V17/V20	<0.001	89	100
Oesophagus	Dmean	0.46	32	57
Spinal Canal	Dmax	<0.001	49	73

*p* < 0.05 indicates statistically significant differences (Wilcoxon signed-rank test).

**Table 3:** Evaluation of intra- and interobserver variability in two patients and two observers for the delineation of structures used in breast radiotherapy and comparison with the performance of commercial software.

	DSC					
	Heart	Lung	Oesophagus	Spinal Canal	PTVbreast	PTVn
Intervariability	0.85	0.99	0.64	0.83	0.92	0.76
Intravariability	0.9	0.99	0.65	0.84	0.92	0.81
PreciseART	0.91	0.95	0.63	0.85	0.91	0.79

Limited to 2 patients; exploratory only, not powered for statistical comparison.

## Data Availability

The data that support the findings of this study are not openly available and are available from the corresponding author upon reasonable request. Data are located in controlled-access data storage at Centre Paul Strauss.

## Supplementary Materials

The following supporting information can be downloaded at: https://www.mdpi.com/article/10.3390/cancers18091386/s1, Supplementary File S1: Definition of geometric metrics used in this study; Supplementary File S2: all the numerical values used in this study.
