# Supplementary material for: Performance of a Vendor System for Systematic Offline Adaptive Breast Helical Radiotherapy
Source: Cancers (Basel). 2026 Apr 27;18(9):1386. doi: 10.3390/cancers18091386 (PMC13163115; doi:10.3390/cancers18091386)
Supplement: Supplementary file 1 [file cancers-18-01386-s001.zip › Supplementary File S1.pdf]

Supplementary File S1 – Definition of geometric metrics used in this study

Metrics based on binary masks

Creation of Binary Masks from Coordinates

A function creates a 3D binary mask for a structure from its contour coordinates.

In the context of CT data, these binary masks are used to represent segmented regions, where each voxel is either 0 (background) or 1 (structure).

Dice Similarity Coefficient (DSC)

The DSC measures the overlap between two binary masks mask1 and mask2:

$$\mathrm{DSC} = \frac{2\,|\mathrm{mask}_1 \cap \mathrm{mask}_2|}{|\mathrm{mask}_1| + |\mathrm{mask}_2|},$$

,

where | · | denotes the number of voxels with a (1) label.

Coordinates Based Metrics

These metrics are computed from sets of surface points A and B extracted directly from RT Struct Dicom file.

Preliminaries: Euclidean Distance in 3D

Let a = (ax, ay, az) and b = (bx, by, bz) be two points in R3. The Euclidean distance between a and b is defined as:

$$d(a,b) = \sqrt{(a_x - b_x)^2 + (a_y - b_y)^2 + (a_z - b_z)^2}.$$

Mean Distance Agreement (MDA)

The MDA is the average of the minimum distances from each point in one set to the other:

$$\mathrm{MDA} = \frac{1}{2}\left(\frac{1}{|A|}\sum_{a\in A}\min_{b\in B} d(a,b) + \frac{1}{|B|}\sum_{b\in B}\min_{a\in A} d(b,a)\right)$$

Hausdorff Distance (HD)

The Hausdorff Distance measures the maximum distance from a point in one set to the nearest point in the other:

$$\mathrm{HD}(A,B) = \max\left\{\sup_{a\in A}\inf_{b\in B} d(a,b),\ \sup_{b\in B}\inf_{a\in A} d(b,a)\right\}$$

95th Percentile Hausdorff Distance (HD95)

To reduce the effect of outliers, the 95th percentile of the distances is used:

$$\mathrm{HD}_{95} = \max\left\{ P_{95}\left(\{\min_{b\in B} d(a,b) : a \in A\}\right),\ P_{95}\left(\{\min_{a\in A} d(b,a) : b \in B\}\right)\right\},$$

where P95 denotes the 95th percentile.

Barycenter and Barycenter Distance

The barycenter (or centroid) of a set of points C (e.g., a segmented region) is computed as:

$$\mathrm{Barycenter}(C) = \left(\frac{1}{N}\sum_{i=1}^{N} x_i,\ \frac{1}{N}\sum_{i=1}^{N} y_i,\ \frac{1}{N}\sum_{i=1}^{N} z_i\right),$$

where N is the number of points in C.

For two sets A and B with barycenters Barycenter(A) = (xA, yA, zA) and Barycenter(B) = (xB, yB, zB), the Euclidean distance between these barycenters is:

$$d\left(\mathrm{Barycenter}(A), \mathrm{Barycenter}(B)\right) = \sqrt{(x_B - x_A)^2 + (y_B - y_A)^2 + (z_B - z_A)^2}.$$
